# Factors Associated With Fatigue in Patients Undergoing Hemodialysis

**DOI:** 10.7759/cureus.22994

**Published:** 2022-03-09

**Authors:** Stavros Tsirigotis, Maria Polikandrioti, Victoria Alikari, Evangelos Dousis, Ioannis Koutelekos, Georgia Toulia, Niki Pavlatou, Georgios I Panoutsopoulos, Dionyssios Leftheriotis, Georgia Gerogianni

**Affiliations:** 1 Department of Nursing, Postgraduate Program "Applied Clinical Nursing", University of West Attica, Athens, GRC; 2 Department of Nutrition and Dietetic Sciences, University of Peloponnese, Kalamata, GRC; 3 Department of Cardiology, Attikon University Hospital of Athens, Athens, GRC

**Keywords:** comorbidity, muscle weakness, tiredness, sleep disorders, depression, fatigue, hemodialysis

## Abstract

Background and objective

Fatigue is frequently experienced by patients undergoing hemodialysis and it has a negative effect on their quality of life. The aim of this study was to investigate the factors associated with fatigue in patients undergoing hemodialysis.

Methods

In this quantitative cross-sectional study, 100 patients on hemodialysis participated. Fatigue was evaluated via the Modified Fatigue Impact Scale (MFIS). Participants also completed a questionnaire about demographics and clinical characteristics. The Kruskal-Wallis test, the Mann-Whitney U test, and Spearman's rho criterion were used to assess the association between fatigue score and patient characteristics. Multiple linear regression was performed to assess the effect of the characteristics on patients' fatigue.

Results

Statistically significant high levels of physical or mental fatigue were found in older patients (p=0.001 and p=0.001), divorced/widowed patients (p=0.001 and p=0.014), those who had children (p=0.019), those who had primary education (p=0.015), those who were not informed about their health problems (p=0.003 and p=0.006), those who had comorbid diseases (p=0.001 and p=0.001), those who believed that regular information did not help to reduce stress (p=0.005 and p=0.004), patients who had insomnia (p=0.001 and p=0.001), patients who felt tired after hemodialysis (p=0.001 and p=0.001), those who thought they had a change in body image (p=0.001 and p=0.001), those who often felt stiffness (p=0.001 and p=0.001), those who sometimes felt nausea (p=0.015 and p=0.038), and those who had limitations in the clothes they could wear (p=0.001 and p=0.001).

Conclusions

The findings of this study showed that physical or mental fatigue had a strong association with advanced age, comorbidities, marital status, level of education, inadequate information about the disease, insomnia, and change in body appearance. Hence, renal professionals need to properly educate dialysis patients on the complicated nature of fatigue in order to manage it effectively and improve their physical, cognitive, and social wellbeing.

## Introduction

Fatigue is commonly experienced by patients undergoing hemodialysis and has a negative effect on their quality of life [1]. Its prevalence rate ranges from 60-97% among hemodialysis patients [2]. These patients suffer from decreased levels of physical activity, low functional capability, and general muscle weakness, which results in a general feeling of fatigue [3]. Mental fatigue is characterized by lack of concentration and inability to remain focused under certain conditions while physical fatigue entails muscle weakness [4].

Symptoms of fatigue are tiredness, weakness, lack of energy [5,6], declined cognitive function, loss of memory, and poor concentration [7], which make patients unable to participate in daily activities. Lack of energy is a source of stress and frustration since patients have limited functional capability [7], which affects their ability to work, look after their families, and achieve personal goals [8]. Thus, fatigue negatively affects their physical, cognitive, and social wellbeing and life satisfaction [2].

Fatigue is a multidimensional problem [9] caused by anemia, uremia, [2], dialysis inadequacy [10], sleep disorders, pain in bones and muscles, poor nutritional status, inflammation, depression, anxiety [5], advanced age, and comorbid diseases [6]. Depression is the most significant psychological factor that is strongly related to fatigue [11]. Similarly, feelings of negative affect, including depressive mood, are reportedly related to significantly high rates of fatigue in dialysis patients [9]. Additionally, it has been found that renal patients who suffer from restless leg syndrome have increased levels of fatigue [12].

However, fatigue among dialysis patients is often unrecognized and untreated [13] since symptoms are usually subjective and not clearly identifiable [14]. In light of this, the aim of this study was to explore the factors associated with fatigue in patients undergoing hemodialysis.

## Materials and methods

Study sample

The study was conducted at a dialysis unit in Athens, Greece, with a convenience sample of 100 patients. The selection criteria for participants were as follows: aged 20 years or older, on hemodialysis for at least three months, and the ability to speak, read, and write Greek. The exclusion criteria were as follows: insufficient language ability, age of more than 85 years, cognitive deterioration, and drug or alcohol abuse. Patients who met the inclusion criteria were informed about the purpose and procedure of the study and confidentiality was assured. Before collecting data, we obtained approval from the Ethics Committee at the Laconia General Hospital (approval number: 9123/29-10-2020). The study was carried out from November 2020 to January 2021.

The Modified Fatigue Impact Scale (MFIS) was utilized for the evaluation of fatigue and a questionnaire that included the following factors were distributed among the participants: (a) demographic characteristics: gender, age, educational level, job, marital status, number of children, (b) clinical characteristics: the degree of awareness of their health problems, comorbid diseases, the stress they felt due to dialysis regimens, whether they desired to receive written information about the management of their condition, pain after hemodialysis, changes in body image, muscle cramps, joint stiffness, nausea, vomiting, and values of urea, creatinine, and phosphorus, (c) information about patients' concerns: changes in social and personal life, changes in the amount of fluid and food intake, fear of the possibility of the shutdown of the hemodialysis machine, difficulties in movement, and concerns about the clothes they could wear. The study was carried out in accordance with the Declaration of Helsinki (1989).

Modified Fatigue Impact Scale (MFIS)

The MFIS is a 21-item self-assessment scale. Participants indicate the fatigue they experienced throughout the last four weeks. Participants circle the corresponding number that best expresses how often their fatigue has affected them in the last four weeks on a 5-point Likert scale. In each of the grades of the 5-point scale, the scores range from 1 to 5. Ten of the 21 questions evaluate the mental fatigue of patients and the other 11 evaluate the physical fatigue of respondents. The scores assigned to the questions are summed up separately for the questions that assess mental fatigue and those that assess physical fatigue, leading to two scores ranging between 10 and 50 for mental fatigue and between 11 and 55 for physical fatigue. Higher scores indicate higher levels of mental and physical fatigue, respectively. The MFIS has high reliability and validity among the Greek population [15].

Statistical analysis

Nominal data are presented as absolute and relative (%) frequencies, while the continuous ones are presented as mean and standard deviation (SD), and median and interquartile range (IQR). The normality of the data was checked with the Kolmogorov-Smirnov criterion and graphically with histograms and Q-Q plots. The Kruskal-Wallis test, the Mann-Whitney U test, and Spearman's rho criterion were used to evaluate the association between fatigue score and patient characteristics. Multiple linear regression was performed to assess the effect of characteristics on patients' fatigue. The results are presented with β regression coefficients and 95% confidence intervals (CI). The observed significance level of 5% was considered statistically significant. All statistical analyses were performed with the SPSS Statistics program version 25 (IBM, Armonk, NY).

## Results

Sample description

A total of 100 patients participated in the study. Most of the participants (75%) were men, while 73% of them were over 60 years old. The majority of the respondents (65%) were married, and pensioners (65%), while 42% had primary education. Most of the participants had two or more children (63%) (Table 1).

**Table 1:** Demographic characteristics of patients (N=100)

Characteristics	Ν (%)
Gender	
Male	75 (75.0%)
Female	25 (25.0%)
Age (years)	
30-40	2 (2.0%)
41-50	9 (9.0%)
51-60	16 (16.0%)
61-70	30 (30.0%)
>70	43 (43.0%)
Marital status	
Single	14 (14.0%)
Married	65 (65.0%)
Divorced	3 (3.0%)
Widowed	17 (17.0%)
Living together	1 (1.0%)
Education level	
Primary school	42 (42.0%)
High school	46 (46.0%)
University	10 (10.0%)
MSc-PhD	2 (2.0%)
Job	
Unemployed	4 (4.0%)
Private employee	3 (3.0%)
Freelancer	16 (16.0%)
Household work	9 (9.0%)
Pensioner	65 (65.0%)
Other	3 (3.0%)
Number of children	
0	14 (14.0%)
1	23 (23.0%)
2	45 (45.0%)
>2	18 (18.0%)

Additionally, 79% of the participants were very or sufficiently informed about their condition of health; 44% suffered from comorbid diseases and 66% had very or sufficient anxiety about the dialysis regimen. Moreover, 36% believed that regular updating was very helpful in reducing stress; 64% wanted to receive written information about management of their disease and 15% stated that they had insomnia. Furthermore, 34% felt tired after the dialysis session, 39% felt more tired at night, and 84% felt tired for a few hours; 37% sometimes felt pain during venous puncture, 73% believed that they had a change in body image, and 34% sometimes had itching. Also, 11% had frequent muscle cramps and 10% had stiffness, while 14% sometimes had nausea and vomiting. The mean values of patients' recent urea, creatinine, and phosphorus were 124, 9, and 6.2, respectively.

Of note, 65% were bothered that they spent a lot of time on hemodialysis, 55% were concerned because they had limited social life due to hemodialysis, and 69% stated that they had experienced changes in their role as a husband/wife. Additionally, 75% stated that they were concerned about the fact that they had to take a limited amount of fluids, and 65% that they should avoid certain foods; 56% were also concerned about the possibility of disruption of arteriovenous anastomosis, and 16% of the possibility of disruption of the dialysis machine. Moreover, 86% stated that they had difficulty in going on vacations and 27% had restrictions on the clothes they could wear. Finally, 31% wanted to hide a part of their body and 70% said that they had a change in their body image.

Regarding the descriptive characteristics of the MFIS scale, the total mean score was 60.7 (SD: 18.0), while for the subscales of Physical and Mental Fatigue, it was 35.1 (SD: 9.9) and 25.5 (SD: 8.7), respectively. The median of the total score was 58.5 (IQR: 49-72.5) while the medians of Physical and Mental Fatigue were 33.5 (IQR: 28-42) and 24 (IQR: 20-31), respectively. At least 50% of patients scored <33.5 (median) and 25% scored <28 in physical fatigue, while 50% had <24 and 25% had <20 in mental fatigue. These values indicate moderate to low levels of patient fatigue.

Association of the fatigue scale with patients' characteristics

Statistically significant associations were found between patients' physical fatigue score and age (p=0.001), marital status (p=0.004), number of children (p=0.019), degree of information about their health problem (p=0.003), whether they had another disease (p=0.001), whether they believed that regular updating helped reduce stress (p=0.005), insomnia (p=0.001), whether they felt tired after hemodialysis, and the duration of fatigue (p=0.001 and p=0.001, respectively), whether they had changes in body image (p=0.001), whether they felt stiffness and nausea/vomiting (p=0.001 and p=0.015, respectively), and whether they felt restricted about what they could wear (p=0.001).

More specifically, patients over the age of 70 had statistically significantly higher levels of physical fatigue (median: 41) than younger patients (median: 31 and 28). Divorced/widowed patients had statistically significantly higher levels of physical fatigue (median: 41) than married (median: 33) and single patients (median: 27). Patients who had children also had higher levels of physical fatigue (median: >34) than those without children (median: 26). Patients who were little or not at all informed about their health problems had higher levels of physical fatigue (median: 41) than those who were very or sufficiently informed (median: 32). Patients who had comorbid diseases had higher levels of physical fatigue (median: 40) than those who did not have comorbid diseases (median: 31). Patients who believed that regular updating did not help reduce stress had statistically significantly high levels of physical fatigue (median: 42). In addition, high levels of physical fatigue were experienced by patients who had insomnia (median: 36), those who felt tired after dialysis (median: 42), those who believed they had a change in their body image (median: 38), those who often felt stiffness (median: 44), those who sometimes felt nausea (median: 37), and those who felt restricted about what they could wear (median: 38) (Table 2).

**Table 2:** Association of physical fatigue scale with patients' characteristics IQR: interquartile range: SD: standard deviation

Variables	Physical fatigue		
	Mean (SD)	Median (IQR)	P-value
Gender			0.325
Male	34.7 (10.3)	32 (26-42)	
Female	36.4 (8.6)	34 (31-42)	
Age (years)			0.001
≤60	29.1 (7.1)	28 (24-32)	
61-70	33.1 (9.1)	31 (28-40)	
>70	40.3 (9.4)	41 (32-49)	
Marital status			0.004
Married/living together	34.7 (9.8)	33 (28-42)	
Single	29.4 (8.8)	27 (24-34)	
Divorced/widowed	40.5 (8.9)	41 (32-47)	
Education level			0.116
Primary school	36.9 (9.6)	36 (29-44)	
High school	34.7 (9.4)	35 (28-42)	
University/MSc-PhD	31.0 (12.1)	28 (22-36)	
Job			0.087
Unemployed/household work	31.5 (6.8)	31 (28-34)	
Employee	32.4 (9.0)	29 (26-36)	
Pensioner	36.9 (10.5)	37 (28-44)	
Number of children			0.019
0	27.9 (7.4)	26 (24-31)	
1	37.1 (11.0)	38 (28-47)	
2	36.4 (8.9)	34 (30-43)	
>2	35.1 (10.7)	36 (26-43)	
Informed about their health problems			0.003
Very	27.8 (11.1)	24 (20-31)	
Enough	35.1 (9.6)	34 (28-42)	
A little/not at all	39.2 (8.3)	41 (32-44)	
Other diseases			0.001
Yes	39.5 (9.4)	40 (32-47)	
No	31.8 (9.0)	31 (25-36)	
Are you nervous about the course of the disease?			0.466
Very	35.3 (9.8)	32 (27-42)	
Enough	33.6 (9.1)	32 (28-41)	
A little/not at all	36.7 (10.8)	36 (28-47)	
Do you believe that regular updating helps reduce stress?			0.005
Very	32.6 (10.8)	30 (25-42)	
Enough	33.9 (7.9)	34 (28-38)	
A little/not at all	40.4 (9.6)	42 (32-49)	
Do you wish to receive written information regarding the management of the disease?			0.104
Yes/sometimes	34.3 (9.4)	32 (28-41)	
No	38.0 (11.3)	41 (30-49)	
How often do you weigh yourself at home?			0.327
Daily	32.7 (8.1)	33 (27-39)	
Every 2-4 days	31.0 (10.5)	32 (22-34)	
Once a week	36.0 (10.1)	36 (28-43)	
Do you have insomnia?			0.001
Yes/sometimes/often	36.5 (10.0)	36 (29-43)	
No	29.9 (7.8)	29 (24-34)	
Do you feel tired after each dialysis session?			0.001
Yes	42.2 (8.5)	42 (37-48)	
Sometimes/often	31.9 (8.4)	31 (26-36)	
When do you feel most tired?			0.236
Morning/noon	35.8 (11.0)	31 (28-43)	
Afternoon	33.0 (9.4)	31 (26-39)	
Night	36.7 (9.5)	36 (29-43)	
What is the duration of fatigue?			0.001
Continuous	47.3 (6.2)	47 (42-54)	
A few hours	33.2 (9.0)	31 (27-40)	
Do you feel pain during venous punctures?			0.194
No	34.2 (10.4)	31 (25-42)	
Rarely	32.0 (8.7)	31 (28-36)	
Sometimes	37.2 (9.8)	37 (31-43)	
Often/yes	36.4 (10.4)	37 (28-44)	
Do you think there has been a change in body image after the diagnosis of the disease?			0.001
Yes	37.6 (9.9)	38 (31-44)	
No	28.4 (6.3)	28 (24-31)	
Have you had itching in your body since you started dialysis?			0.057
No	32.8 (9.3)	31 (26-38)	
Rarely/sometimes	35.6 (9.8)	34 (28-43)	
Often /yes	39.8 (10.6)	41 (32-48)	
Do you have muscle cramps?			0.710
No	35.1 (10.0)	36 (28-43)	
Rarely/sometimes	34.7 (9.8)	32 (28-41)	
Yes/often	37.1 (10.7)	39 (29-44)	
Do you have stiffness in your joints?			0.001
No	28.0 (6.3)	28 (24-32)	
Rarely/sometimes	35.3 (9.0)	33 (29-42)	
Yes/often	44.4 (7.1)	44 (41-51)	
Do you feel nauseous and vomiting?			0.015
No	33.1 (9.7)	31 (26-40)	
Rarely/sometimes	38.1 (9.9)	37 (31-48)	
Are you concerned about the fact that you now have a limited social life compared to what you had before you started dialysis?			0.967
Yes/sometimes	35.0 (9.4)	34 (28-42)	
No	35.9 (13.4)	31 (24-49)	
Are there any changes in your role as a husband/wife?			0.686
Yes/sometimes	34.9 (9.4)	33 (28-42)	
No	36.4 (12.0)	41 (24-49)	
Are you worried about the possibility of the dialysis machine shutting down?			0.901
Yes/sometimes	34.9 (8.7)	34 (28-41)	
No	35.4 (11.2)	31 (27-44)	
Do you have any restrictions about the clothes you can wear?			0.001
Yes/sometimes	37.9 (9.3)	38 (31-44)	
No	30.3 (9.2)	28 (24-35)	
Do you want to hide or conceal your body or any part of your body? (like the fistula)			0.345
Yes/sometimes	35.9 (9.1)	34 (29-42)	
No	34.3 (10.7)	32 (25-42)	
	Spearman’s rho	P-value	
Recent urea	-0.004	0.971	
Recent creatinine	-0.078	0.438	
Recent phosphorus	0.040	0.696	

Statistically significant associations were found between the score of patients' mental fatigue and age (p=0.001), marital status (p=0.014), educational level (p=0.015), degree of information about their health problem (p=0.006), whether they had another disease (p=0.001), whether they believed that regular updating helps reduce stress (p=0.004), insomnia (p=0.001), whether they felt tired after hemodialysis, and the duration of fatigue (p=0.001 and p=0.001, respectively), whether they had changes in body image (p=0.001), whether they felt stiffness and nausea/vomiting (p=0.001 and p=0.038, respectively), and whether they felt restricted about what they could wear (p=0.001).

More specifically, patients over the age of 70 years had statistically significantly higher levels of mental fatigue (median: 30) than younger patients (median: 22 and 20). Divorced/widowed patients had statistically significantly higher levels of mental fatigue (median: 30) than married (median: 22) and singles (median: 24) patients. Patients with primary education had higher levels of mental fatigue (median: 28) than those with secondary (median: 22) and university level (median: 20) education. Patients who were little or not at all informed about their health problems had higher levels of mental fatigue (median: 29) than those who were very or sufficiently informed (median: 11). Patients with comorbid diseases had higher levels of mental fatigue (median: 27) than those without comorbid diseases (median: 22). Patients who believed that regular information did not help reduce stress had statistically significantly more mental fatigue (median: 30). In addition, higher levels of mental fatigue were experienced by patients who had insomnia (median: 27), those who felt tired after dialysis (median: 30), and those who experienced persistent fatigue (median: 32), those who thought that they had a change in their body image (median: 28), those who often felt stiffness (median: 31), and those who sometimes felt nausea (median: 29), and those who felt restricted about what they could wear (median: 28) (Table 3).

**Table 3:** Association of mental fatigue scale with patients' characteristics IQR: interquartile range: SD: standard deviation

Variables	Mental fatigue		
	Mean (SD)	Median (IQR)	P-value
Gender			0.678
Male	25.7 (9.1)	24 (20-31)	
Female	25.2 (7.3)	22 (20-30)	
Age (years)			0.001
≤60	21.3 (6.3)	20 (17-26)	
61-70	22.8 (8.2)	22 (20-28)	
>70	30.1 (8.2)	30 (23-36)	
Marital status			0.014
Married/living together	24.8 (8.1)	22 (20-30)	
Single	21.8 (8.6)	24 (13-29)	
Divorced/widowed	30.5 (9.0)	30 (26-38)	
Education level			0.015
Primary school	27.7 (7.7)	28 (22-32)	
High school	24.9 (8.7)	22 (20-30)	
University/MSc-PhD	20.2 (9.9)	20 (11-24)	
Job			0.277
Unemployed/household work	24.7 (6.6)	22 (20-31)	
Employee	23.3 (7.0)	22 (20-26)	
Pensioner	26.6 (9.4)	27 (20-32)	
Number of children			0.399
0	21.5 (8.5)	23 (13-29)	
1	27.2 (9.5)	26 (20-32)	
2	26.2 (8.0)	24 (20-30)	
>2	25.1 (9.0)	24 (19-31)	
Informed about their health problems			0.006
Very	18.3 (11.0)	11 (10-28)	
Enough	25.5 (7.7)	24 (20-31)	
A little/not at all	29.4 (8.2)	29 (26-32)	
Other diseases			0.001
Yes	28.3 (8.8)	27 (22-33)	
No	23.3 (8.0)	22 (18-30)	
Are you nervous about the course of the disease?			0.426
Very	24.0 (7.0)	22 (20-29)	
Enough	25.0 (7.3)	24 (20-30)	
A little/not at all	27.4 (10.9)	28 (20-36)	
Do you believe that regular updating helps reduce stress?			0.004
Very	23.3 (8.6)	21 (19-31)	
Enough	24.1 (6.6)	23 (20-28)	
A little/not at all	30.7 (9.6)	30 (26-38)	
Do you wish to receive written information regarding the management of the disease?			0.081
Yes/sometimes	24.5 (7.6)	23 (20-30)	
No	29.0 (11.1)	30 (19-38)	
How often do you weigh yourself at home?			0.051
Daily	20.9 (7.3)	20 (16-27)	
Every 2-4 days	21.4 (10.5)	21 (10-26)	
Once a week	26.9 (8.4)	26 (20-32)	
Do you have insomnia?			0.001
Yes/sometimes/often	27.2 (8.5)	27 (21-32)	
No	19.3 (6.3)	20 (16-23)	
Do you feel tired after each dialysis session?			0.001
Yes	30.3 (9.1)	30 (25-36)	
Sometimes/often	23.4 (7.3)	22 (20-29)	
When do you feel most tired?			0.472
Morning/noon	26.6 (9.5)	24 (20-33)	
Afternoon	23.8 (7.4)	23 (20-30)	
Night	26.5 (9.2)	27 (20-31)	
What is the duration of fatigue?			0.001
Continuous	34.5 (9.0)	32 (30-41)	
A few hours	24.0 (7.9)	23 (20-30)	
Do you feel pain during venous punctures?			0.146
No	25.4 (8.8)	23 (20-32)	
Rarely	22.5 (6.9)	22 (20-26)	
Sometimes	27.9 (9.0)	28 (21-33)	
Often/yes	24.4 (9.2)	26 (17-30)	
Do you think there is a change in body image after the diagnosis of the disease?			0.001
Yes	27.3 (8.8)	28 (21-32)	
No	20.7 (6.2)	20 (17-24)	
Have you had itching in your body since you started dialysis?			0.258
No	23.9 (6.8)	22 (20-30)	
Rarely/sometimes	26.0 (9.4)	26 (20-31)	
Often/yes	28.5 (10.1)	29 (20-35)	
Do you have muscle cramps?			0.622
No	24.5 (7.0)	23 (21-30)	
Rarely/sometimes	25.6 (9.0)	25 (20-31)	
Yes/often	27.6 (10.4)	28 (22-35)	
Do you have stiffness in your joints?			0.001
No	21.1 (6.8)	21 (17-26)	
Rarely/sometimes	25.5 (8.9)	24 (20-30)	
Yes/often	31.5 (7.0)	31 (25-39)	
Do you feel nauseous and vomiting?			0.038
No	23.8 (7.6)	22 (20-29)	
Rarely/sometimes	28.3 (10.0)	29 (20-36)	
Are you concerned about the fact that you now have a limited social life compared to what you had before you started dialysis?			0.157
Yes/sometimes	24.9 (7.9)	23 (20-30)	
No	29.6 (12.3)	28 (24-35)	
Are there any changes in your role as a husband/wife?			0.103
Yes/sometimes	24.7 (7.7)	23 (20-30)	
No	28.9 (11.7)	30 (17-38)	
Are you worried about the possibility of the dialysis machine shutting down?			0.356
Yes/sometimes	24.7 (7.3)	24 (20-29)	
No	26.5 (10.0)	24 (20-33)	
Do you have any restrictions on the clothes you can wear?			0.001
Yes/sometimes	27.2 (7.8)	28 (22-32)	
No	22.6 (9.4)	20 (17-26)	
Do you want to hide or conceal your body or any part of your body? (like the fistula)			0.422
Yes/sometimes	26.0 (7.4)	26 (20-31)	
No	25.0 (10.0)	23 (19-30)	
	Spearman’s rho	P-value	
Recent urea	0.076	0.454	
Recent creatinine	-0.026	0.795	
Recent phosphorus	-0.031	0.763	

Effect of patients' characteristics on the fatigue scale

Multiple linear regression was performed to assess the effect of patient characteristics (independent factors) on the fatigue they experienced (dependent variable).

Regarding physical fatigue, we observed that patients who sometimes felt tired after dialysis had a statistically significantly lower score of 4.8 points compared to patients who felt tired after each session (β=-4.81, 95% CI: -8.78 to -0.83, p=0.018). In addition, patients who felt tired for a few hours had a statistically significantly lower score of 6.5 points compared to those who felt continuous fatigue (β=-6.53, 95% CI: -11.91 to -1.15, p=0.018). Also, patients who often felt joint stiffness and those who sometimes felt nauseous had 7 and 3.7 points, respectively, which were higher scores for physical fatigue compared to those who did not feel stiffness and nausea (β=7.08, 95% CI: 1.64-12.53, p=0.012 and β=3.67, 95% CI: 0.18-7.17, p=0.040, respectively).

Regarding mental fatigue, we observed that patients who did not have insomnia had a statistically significantly lower score of mental fatigue at 6.3 compared to patients who had insomnia (β=-6.31, 95% CI: -10.01 to -2.61, p=0,001). In addition, patients who felt tired for a few hours had a statistically significantly lower score for mental fatigue 8 points than those who felt constant fatigue (β=-8.04, 95% CI: -13.01 to -3.07, p=0.002). In addition, patients who sometimes felt nauseous had a higher score of mental fatigue at 5 points than those who did not (β=5.01, 95% CI: 1.62-8.40, p=0.004) (Table 4).

**Table 4:** Effect of patients' characteristics on the fatigue scale

Variables	Physical fatigue		Mental fatigue	
	β coefficient (95% CI)	P-value	β coefficient (95% CI)	P-value
Age (years)				
≤60	Ref. Cat.		Ref. Cat.	
61-70	-0.09 (-4.02-3.83)	0.962	-0.48 (-4.48-3.53)	0.814
>70	3.82 (-0.29-7.93)	0.068	3.41 (-0.68-7.50)	0.101
Status				
Married/living together	Ref. Cat.		Ref. Cat.	
Single	2.32 (-10.80-15.43)	0.726	-0.70 (-5.13-3.74)	0.755
Divorced/widowed	0.27 (-3.72-4.26)	0.893	1.46 (-2.47-5.39)	0.461
Education level				
Primary school	-		Ref. Cat.	
High school	-		1.95 (-1.14-5.04)	0.213
University/MSc-PhD	-		0.80 (-5.51-7.10)	0.802
Number of children				
0	Ref. Cat.		-	
1	6.10 (-7.52-19.72)	0.375	-	
2	6.51 (-6.72-19.74)	0.33	-	
>2	2.99 (-10.48-16.46)	0.659	-	
Informed about their health problems				
Very	Ref. Cat.		Ref. Cat.	
Enough	3.53 (-1.66-8.72)	0.18	5.82 (-0.69-12.34)	0.079
A little/not at all	1.34 (-5.39-8.07)	0.692	3.37 (-4.48-11.21)	0.395
Other diseases				
Yes	Ref. Cat.		Ref. Cat.	
No	2.49 (-0.84-5.82)	0.14	2.53 (-0.73-5.78)	0.126
Do you believe that regular updating helps reduce stress?				
Very	Ref. Cat.		Ref. Cat.	
Enough	-1.91 (-5.41-1.59)	0.28	-1.77 (-5.06-1.52)	0.286
A little/not at all	4.23 (-1.16-9.62)	0.122	4.90 (-0.29-10.09)	0.064
Do you have insomnia?				
Yes/sometimes/often	Ref. Cat.		Ref. Cat.	
No	-1.93 (-5.77-1.91)	0.321	-6.31 (-10.01 to -2.61)	0.001
Do you feel tired after each dialysis session?				
Yes	Ref. Cat.		Ref. Cat.	
Sometimes/often	-4.81 (-8.78 to -0.83)	0.018	-1.75 (-5.63-2.12)	0.369
What is the duration of fatigue?				
Continuous	Ref. Cat.		Ref. Cat.	
A few hours	-6.53 (-11.91 to -1.15)	0.018	-8.04 (-13.01 to -3.07)	0.002
Do you think there is a change in body image after the diagnosis of the disease?				
Yes	Ref. Cat.		Ref. Cat.	
No	-2.44 (-6.78-1.90)	0.266	-1.89 (-5.93-2.16)	0.355
Do you have stiffness in your joints?				
No	Ref. Cat.		Ref. Cat.	
Rarely/sometimes	0.25 (-4.24-4.74)	0.913	-1.15 (-5.42-3.11)	0.591
Yes/often	7.08 (1.64-12.53)	0.012	1.13 (-4.16-6.42)	0.672
Do you feel nauseous and vomiting?				
No	Ref. Cat.		Ref. Cat.	
Rarely/sometimes	3.67 (0.18-7.17)	0.04	5.01 (1.62-8.40)	0.004

## Discussion

The present study found that patients over the age of 70 years and those with comorbid diseases had statistically significantly high levels of physical or mental fatigue. It is well known that patients of advanced age have decreased levels of physical functioning due to comorbid diseases, which lead to complications, disabilities [16], and loss of energy [17]. Given that the prevalence of renal failure is increasing in individuals over 65 years old, it is understandable that elderly people need closer attention. They frequently have to overcome several challenges such as the complexity of therapy, the severity of the disease, and greater effort to follow the therapeutic regimen [18].

Fatigue in patients with advanced age is a multifactorial problem due to depression, anxiety, and subjective sleep quality. Furthermore, illness perception, coping mechanisms, and self-efficacy are gradually deteriorating in them. An equally important factor is that the elderly are frequently unable to adhere to treatment, which leads to the deterioration of their physical state [17]. Meanwhile, loss of skeletal muscle, which constitutes the largest type of tissue mass and accounts for 40-45% of total body weight, leads to functional failure, resulting in poor outcomes, especially in elderly individuals [19]. However, fatigue is often under-recognized and under-treated by healthcare providers as its symptoms are often not visible. Healthcare professionals frequently attribute fatigue to the advanced age or side effects of hemodialysis [20]. At the same time, age is one of the strongest predictors of depression among hemodialysis patients, which may explain the fatigue in the elderly to some extent [21].

Moreover, this study showed that divorced/widowed patients had statistically significantly higher levels of physical or mental fatigue than married and single patients. A possible explanation for this finding is that patients who lead their lives without support are exposed to worse clinical outcomes. It is well documented that social support consists of a modifiable psychosocial factor of significant importance for survival. The need for social support varies among patients undergoing hemodialysis according to the quality and quantity of their social network or the severity of the disease [22]. It is important to take into account that patients undergoing long-term hemodialysis usually face problems with marital relationships since spouses feel emotional and psychological distress due to problems arising from hemodialysis [23].

It can be assumed that this vulnerable group of patients (no spouse/pattern) are deprived of the benefits associated with social support. Social support improves the quality of life through various mechanisms such as increasing patients’ satisfaction from the provided care, enhancing adherence to the therapeutic regimen including diet and fluid restrictions, or alleviating symptoms of fatigue. This finding may provide guidance to the healthcare providers, family members, and social services about the importance of social support in dialysis patients [22].

The present study also found that patients with primary education and those who were little or not at all informed about their health problems had statistically significantly high levels of physical or mental fatigue. The association with the level of education can be viewed in the context of patients’ capability to understand health-related information. Patients’ knowledge is regarded as an important factor in the management of their disease since those who are knowledgeable understand their condition better and comply with the restrictions of their treatment [16]. However, individuals with low education levels have more difficulty in accepting or comprehending recommendations related to the therapeutic regimen, disease management, and necessary alterations in everyday living. Possibly, these individuals fail to recognize the importance of alleviating fatigue [24]. The mechanism by which low levels of education are linked to fatigue is complex. Possibly, these patients are less likely to have access to healthcare or have difficulty retrieving health information due to a lack of understanding. They may also experience difficulties in learning self-care skills, which leads to exacerbations and a higher burden of symptoms [25,26].

The present study also found that patients who had insomnia had statistically significantly high levels of physical or mental fatigue. These findings are in line with those of a previous study [27]. It can be assumed that sleep disorders have a strong relationship with fatigue since they lead to daytime sleepiness, poor concentration, and increased levels of inflammatory cytokines in patients’ blood [10]. Additionally, the findings of this study showed that patients who had a change in body image had statistically significantly high levels of physical or mental fatigue. Body image is one of the most stressful factors for patients on hemodialysis, often affecting their psychological status. Changes in body image after the initiation of dialysis can be caused by weight loss, muscle wasting, changes in skin color, and marks caused by venous puncture [28].

The results of this study offer important information to renal professionals about the factors related to fatigue in patients on hemodialysis. The findings of this study indicated that fatigue had a strong relationship with advanced age, comorbidity, marital status, level of education, inadequate information about the disease, insomnia, and changes in body appearance. Therefore, this study highlights the importance of early diagnosis of fatigue by renal professionals and the provision of the appropriate information to dialysis patients about the complicated nature of fatigue.

## Conclusions

Fatigue is a common problem among hemodialysis patients, leading to poor quality of life. The findings of the present study indicated high levels of fatigue in patients aged over 70 years, those with comorbidities, divorced/widowed patients, those with primary education, those having inadequate information about the disease, and patients with insomnia and changes in body image. Renal health professionals need to educate patients about the symptoms of fatigue and help them combat it effectively in order to improve their physical, cognitive, and social wellbeing.

## Appendices

Modified Fatigue Impact Scale (MFIS)

**Table 5:** Modified Fatigue Impact Scale (MFIS)

1. I have been less alert		0	1	2	3	4
2. I have had difficulty paying attention for long periods of time		0	1	2	3	4
3. I have been unable to think clearly		0	1	2	3	4
4. I have been clumsy and uncoordinated		0	1	2	3	4
5. I have been forgetful		0	1	2	3	4
6. I have had to pace myself in my physical activities		0	1	2	3	4
7. I have been less motivated to do anything that requires physical effort		0	1	2	3	4
8. I have been less motivated to participate in social activities		0	1	2	3	4
9. I have been limited in my ability to do things away from home		0	1	2	3	4
10. I have trouble maintaining physical effort for long periods		0	1	2	3	4
11. I have had difficulty making decisions		0	1	2	3	4
12. I have been less motivated to do anything that requires thinking		0	1	2	3	4
13. My muscles have felt weak		0	1	2	3	4
14. I have been physically uncomfortable		0	1	2	3	4
15. I have had trouble finishing tasks that require thinking		0	1	2	3	4
16. I have had difficulty organizing my thoughts when doing things at home or at work		0	1	2	3	4
17. I have been less able to complete tasks that require physical effort		0	1	2	3	4
18. My thinking has been slowed down	0		1	2	3	4
19. I have had trouble concentrating	0		1	2	3	4
20. I have limited my physical activities	0		1	2	3	4
21. I have needed to rest more often or for longer periods	0		1	2	3	4
